# Implementation of large, multi-site hospital interventions: a realist evaluation of strategies for developing capability

**DOI:** 10.1186/s12913-024-10721-w

**Published:** 2024-03-06

**Authors:** Janet C Long, Natalie Roberts, Emilie Francis-Auton, Mitchell N Sarkies, Hoa Mi Nguyen, Johanna I Westbrook, Jean-Frederic Levesque, Diane E Watson, Rebecca Hardwick, Kate Churruca, Peter Hibbert, Jeffrey Braithwaite

**Affiliations:** 1 Australian Institute of Health Innovation, Macquarie University, Sydney, Australia; 2 Centre for Primary Health Care and Equity, University of New South Wales, Kensington, NSW Australia; 3 Agency for Clinical Innovation, St Leonards, NSW Australia; 4 Bureau of Health Information, St Leonards, NSW Australia; 5 Peninsula Medical School, Faculty of Health, University of Plymouth, Plymouth, UK

**Keywords:** Implementation, Change management, Learning culture, Capacity development

## Abstract

**Background:**

This study presents guidelines for implementation distilled from the findings of a realist evaluation. The setting was local health districts in New South Wales, Australia that implemented three clinical improvement initiatives as part of a state-wide program. We focussed on implementation strategies designed to develop health professionals’ capability to deliver value-based care initiatives for multisite programs. Capability, which increases implementers’ ability to cope with unexpected scenarios is key to managing change.

**Methods:**

We used a mixed methods realist evaluation which tested and refined program theories elucidating the complex dynamic between context (C), mechanism (M) and outcome (O) to determine what works, for whom, under what circumstances. Data was drawn from program documents, a realist synthesis, informal discussions with implementation designers, and interviews with 10 key informants (out of 37 identified) from seven sites. Data analysis employed a *retroductive* approach to interrogate the causal factors identified as contributors to outcomes.

**Results:**

CMO statements were refined for four initial program theories: *Making it Relevant–* where participation in activities was increased when targeted to the needs of the staff; *Investment in Quality Improvement–* where engagement in capability development was enhanced when it was valued by all levels of the organisation; *Turnover and Capability Loss–* where the effects of staff turnover were mitigated; and *Community-Wide Priority–* where there was a strategy of spanning sites. From these data five guiding principles for implementers were distilled: (1) Involve all levels of the health system to effectively implement large-scale capability development, (2) Design capability development activities in a way that supports a learning culture, (3) Plan capability development activities with staff turnover in mind, (4) Increased capability should be distributed across teams to avoid bottlenecks in workflows and the risk of losing key staff, (5) Foster cross-site collaboration to focus effort, reduce variation in practice and promote greater cohesion in patient care.

**Conclusions:**

A key implementation strategy for interventions to standardise high quality practice is development of clinical capability. We illustrate how leadership support, attention to staff turnover patterns, and making activities relevant to current issues, can lead to an emergent learning culture.

**Supplementary Information:**

The online version contains supplementary material available at 10.1186/s12913-024-10721-w.

## Introduction

Significant variation exists worldwide in the provision and utilisation of medical resources, and resultant care outcomes within and across health systems [1]. These differences have been seen in variable approaches to treatment and disease management, including diagnosis and prescribing processes, as well as the availability of physicians and inpatient resources [2, 3]. Variation in care may be attributable to differences in population characteristics and preferences, such as age, socioeconomic status, and prevalence of disease [2, 4]. However, this variation becomes unwarranted when it reflects differences in individual health professional, team or health system performance, indicating inconsistencies in the quality of care delivery [2]. To address this, health systems may employ interventions to reduce unwarranted variation in care, such as use of clinical practice guidelines or standardisation of clinical pathways [5]. Large-scale initiatives seek to standardise practice at scale by implementing a standard, evidence-based care approach across multiple organisations. Implementing improvement across the whole system increases efficiency and quality of care resulting in population-level outcomes [6]. A key implementation strategy to enable change in this setting is capability development.

### Capability development

Within healthcare, significant attention is placed on continuing professional development, with a focus on competency, or the capacity to apply what is known in a specific circumstance [7, 8–10]. Competencies, such as registration requirements and performance assessments, are widely utilised in practice-based industries to establish benchmarks for performance and practice [9]. However, as competency reflects the ability to meet known challenges, competencies alone may not fulfill the demands required of the unfamiliar or unpredictable scenarios encountered in healthcare [9], or handle changes in practice, work flow and team processes in response to improvement interventions. As a result, recent attention has shifted towards developing capability within healthcare [9, 11]. Developing capability involves moving beyond clinical competency, to the ability to respond flexibly, navigate change, promote a learning culture, and adapt to new circumstances [9, 12, 13]. Capability development initiatives, such as frameworks [14] and deliberate learning activities, have been implemented across all levels of the health system to improve care [11, 15]. 

Capability is developed across organisations in an iterative process [16]. That is, by engaging the relevant clinical, managerial and governing levels in a dialogue that refines and distributes knowledge gained via experience and external successes [16, 17]. Consequently, capability development efforts may be impacted by organisational features at the macro-, meso-, and micro-level, including organisational culture, leadership, resource allocation, time constraints and the ability for individuals to develop new skills [16, 17]. For example, senior managers and decision makers at the macro level may not adequately consider the factors impacting implementation for clinical staff at the micro level (e.g., suitable resources, system integration), while processes established at the micro level (e.g., limited review of feedback from audits) may hinder the decision-making of agents at the macro-level [16]. As a result of these barriers in integration, change efforts within levels may or may not translate to system-wide improvements in care [16]. 

### Realist studies

Realist studies offer insights into the reasons why the implementation of initiatives produce desired or undesired results, by examining the interrelationships between key contextual factors, the mechanisms these factors give rise to, and their outcomes [18]. For capability development strategies, realist studies are well placed to provide nuanced data on what must be present to lead to positive outcomes especially across macro, meso and micro levels as described here. An example of a realist approach in a large-scale health system transformation comes from Greenhalgh and colleagues’ evaluation exploring improvement work across two adjacent health services in London [19]. They identified six key mechanisms driving change, and the relevant contextual factors involved in triggering those mechanisms to produce the observed outcomes. For example, integrating IT services across providers was achieved (desirable outcome) when supported by infrastructure (context) that facilitated connections between organisations (mechanism), whereas integration was constrained (undesirable outcome) when infrastructure was suited to siloed working (context that failed to trigger connections) [19]. Employing a realist approach disentangled the complex roles and contributions of unique contextual factors, and agents at macro-, meso- and micro-levels in a large-scale health system transformation.

The aim of this study was to use a realist approach to collect data to confirm, refute, or refine hypothesised contexts, mechanisms and outcome statements related to capability development strategies used in the implementation of large-scale value-based healthcare initiatives. These data were then distilled into guiding principles to guide implementers and developers of future initiatives.

### Setting

**Fig. 1:**
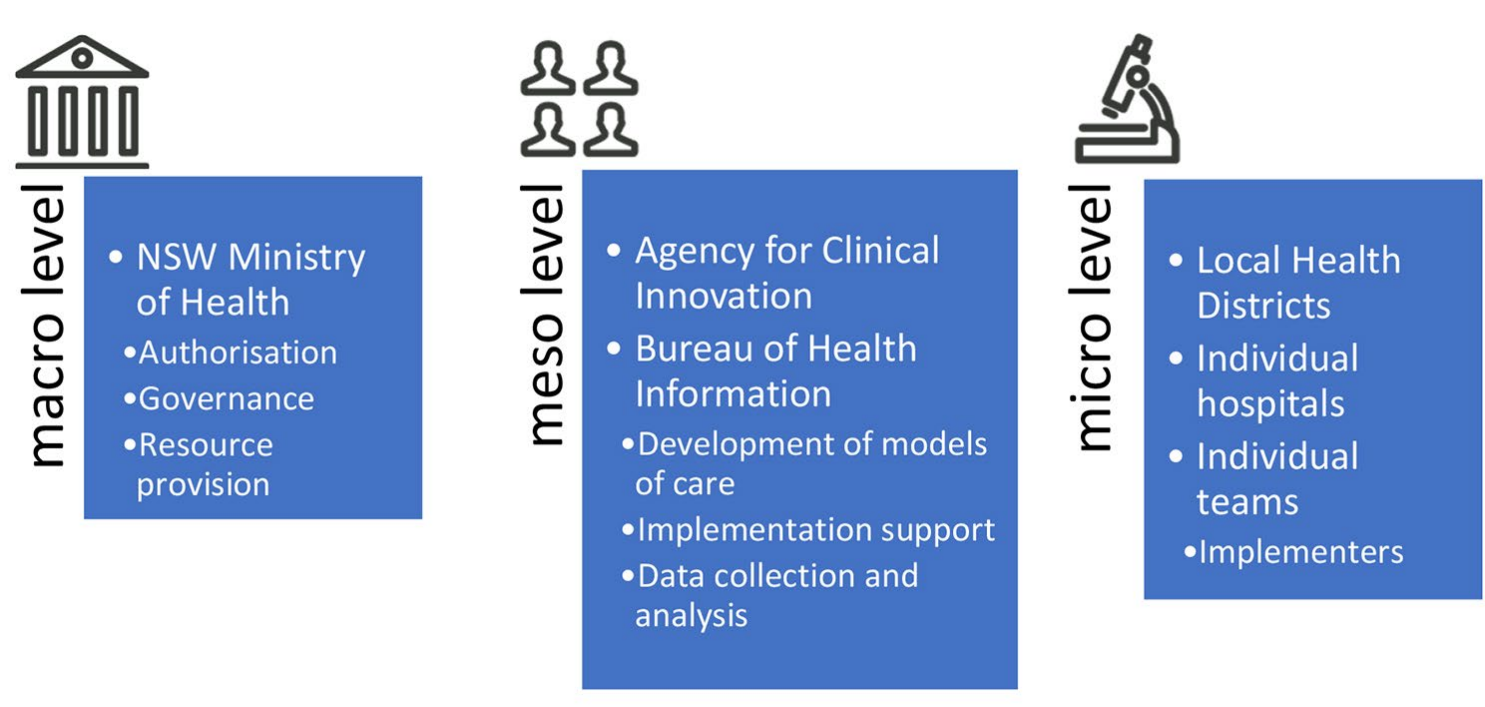
Conceptualisation of levels within the health system involved in the Leading Better Value Care initiatives

This paper reports results of a realist evaluation examining the implementation of three Leading Better Value Care initiatives (LBVC) to all 16 local health districts in New South Wales (NSW), Australia. The initiatives were designed, authorised and funded by the NSW Ministry of Health at the macro-level, and models of care developed and implementation support led by the Agency for Clinical Innovation (ACI), and data monitoring support by the Bureau of Health Information at the meso-level. The implementers themselves — (i.e., the hospital-based clinical teams enacting change) were conceptualised as working at the micro-level (Fig. 1). This paper will discuss implementation strategies that sought to develop capability around three programs targeting inpatient care of people with (1) chronic heart failure (CHF) (2) chronic obstructive pulmonary disease (COPD), and (3) diabetes mellitus (DM), (see Table 1). These three programs were chosen as they were implemented in a similar way and were in the same setting, i.e., inpatient wards of acute hospitals. All initiatives in the LBVC program followed a similar implementation pathway. They were authorised by the NSW Ministry of Health and widely communicated as a state-wide priority. Funding was given to the local health districts to support additional staff and resources required for initial implementation, and data on clinical outcomes and processes were monitored and made available to implementers to build a tension for change and to track progress. Support for the initiatives was given by project officers from the NSW Agency for Clinical Innovation. All three inpatient programs sought to reduce unwarranted variation in practice by developing capability, encouraging local education and skills development around published clinical practice guidelines, and a review of current processes and documentation to support this.

**Table 1:** The three Leading Better Value Care Initiatives studied, patient groups and their aims

Initiative	Patient population	Aim
Chronic heart failure (CHF)	People aged over 18 years, admitted with symptoms suggestive of chronic heart failure	Aims to reduce 28-day readmission and 30-day mortality by a focus on reducing unwarranted variation from best practice, enhance prevention, improve the management and mitigation of risks for people with chronic heart failure.
Chronic obstructive pulmonary disease (COPD)	Acute admitted patients aged 40 years and over with COPD	Aims to reduce 28-day readmission and 30-day mortality by a focus on reducing unwarranted clinical variation and optimisation of lung function for people with COPD.
Inpatient management of diabetes mellitus (DM)	Acute admitted patients aged 16 years and over with diabetes requiring subcutaneous insulin management	Aims to reduce the length of hospital admission for people with diabetes requiring subcutaneous insulin by optimising glucose management.

## Methods

### Theoretical framework and design

This study employed a realist approach to understand large-scale systems change. Grounded in realism, realist approaches explain why interventions work well in some circumstances, or for some people, but not others by making explicit the theories of the causes or drivers of change [18]. Program theories hypothesise the drivers of change by configuring the relationships between contexts, mechanisms and outcomes impacting program implementation [20]. 

All methods were carried out in accordance with relevant guidelines and regulations. Data collection and analysis occurred in three stages and has been outlined in detail in our protocol paper and a methods paper [21, 22]. Briefly, Stage 1 involved team-based discussions which we have termed a *realist dialogic approach*, [21] where the research team developed initial program theories which were expressed as context-mechanism-outcome (CMO) statements (e.g., “When clinical staff are given quarantined time for capability development activities (C), they feel respected and see the value of the ‘investment’ (M), leading to greater commitment to the activities, and ownership of the initiative (O)”). Team discussions drew on a literature review [23], the research team’s knowledge of middle-range theories, and informal discussions with key stakeholders at the NSW Ministry of Health (*n* = 2) and the NSW Agency for Clinical Innovation (*n* = 5).

In Stage 2, the initial program theories were tested and refined using realist interviews with key informants from the NSW Ministry of Health, ACI and implementers from the NSW-based hospital sites. The interview schedule developed from the hypothesised CMOs and divided into the eight initial program theory areas (e.g., one schedule for CMOs about leadership, another for CMOs about data monitoring). Questions were tailored to focus closely on the hypothesised CMOs and asked detailed questions about specific contexts, mechanisms and outcomes observed or experienced by the interviewees. Interviewees were therefore matched to the initial program theory area they would know most about (e.g., clinical staff at the micro level were best placed to describe capability development while meso level implementation support staff were good informants for cross-site collaboration). Retroductive analysis was undertaken on the interview data [24]. Interpretation of data was aided by several other pieces of work that were conducted in parallel: a realist synthesis on implementation of large-scale improvement initiatives [25] which included 51 articles, and a review of 126 publicly available LBVC implementation documents. Finally, Stage 3 involved the distillation of guiding principles for designers and implementers of large-scale initiatives, based on the supported and refined hypotheses. Details of these stages are given below. We note that this work contributed to, but was not the final stage outcome described in our protocol as “ the development of generalisable theoretical models.” [22].

### Recruitment

The LBVC program’s state-wide steering committee identified eligible participants from local health districts and invited them to an interview on our behalf. Participants were knowledgeable about one or more initiatives at one or more sites (i.e., some were knowledgeable about a single site and a single initiative, others were single initiative leaders across all hospitals in their health district, while others had oversight and knowledge of all LBVC initiatives across all hospitals in their district). An Information Sheet was provided to participants, identifying the interviewers as an independent research team, and assuring them their comments would not be identifiable at the individual or local health district/site level. Participants gave formal verbal consent at the start of the interview. We interviewed all participants who accepted the invitation and we were satisfied with the mix of sites and roles this gave.

### Data collection

Semi-structured realist interviews were held remotely over Zoom, Skype or telephone between November 2020– August 2021. Access to participants was difficult during COVID-19 response and increased pressure on health services staff limited their participation to some extent. The interviews were conducted by two experienced qualitative researchers (MS, EFA) and lasted 30 to 60 min. The interview guides were developed from the initial program theories with the aim to confirm, refute or refine the hypothesised CMO statements. Specific aspects of capability covered were: (i) improvement of clinicians’ knowledge and skills, (ii) translation of those knowledge and skills into practice change, (iii) development of a quality improvement culture, (iv) establishing the initiatives as a community-wide priority, (v) sharing or receiving advice on implementation from other NSW sites. Interviews were audio recorded and transcribed verbatim. Data were deidentified prior to analysis and reporting. Interview guide is shown in Supplementary File 1.

### Data analysis

Interview data was imported into NVivo20 for analysis. Data were coded as having relevance to one or more of the hypothesised CMO statements or as raising issues that could form a new statement. Exemplar quotes were selected to support, refute or refine the CMO statements. Interpretation of the data was aided by conceptual understandings of large scale change from the realist synthesis [25] (e.g., unique aspects of top down rather than grass-roots approaches).

In the final stage, recognising that realist results have somewhat limited utility for implementers due to the very specific nature of the CMOs, we distilled findings to produce principles to guide capability development strategies for the implementation of future large-scale initiatives. The procedure for this was similar to the dialogic approach used to develop the initial program theory areas, in that it was based on reflection, discussion and drew on a range of data sources [26]. The core research team read and reread the final set of CMO statements, pulling out themes and recurring ideas, e.g., grouping CMOs pertinent to an aspect of authorisation. Next, the team spent time reflecting on key points of each theme, informed by their own clinical experience (to fill in the gaps on tacit knowledge underlying constructs), findings of the realist synthesis (e.g., concepts related to implementation across multiple sites, CMOs developed from, and confirmed by the literature), information received via review of program documents and initial informal chats with partners overseeing and effecting the implementation, and the team’s knowledge of implementation theory and practice. These were crafted into statements or principles which were then discussed with the broader research team and partners, and then refined.

## Results

### Participants

Ten informants across seven local health districts completed interviews on the role of capability development in the implementation of the CHF, COPD and DM initiatives. Participants occupied a variety of roles, including education, project leads, as well as strategic and managerial positions across whole districts.

### Capability development strategies used

All sites received audit results for a range of indicators. Rather than a prescribed list of actions, individual sites were given flexibility to design their capability development activities based on the published guidelines. For example, across one health district, nurses completed surveys to assess staff’s knowledge of CHF and DM to inform the scope and focus of nurse practitioner-led education workshops. These workshops focused on patient self-management and medication delivery, to support patients throughout their health journey. Similar education programs were implemented for COPD, where nurses completed smoking cessation programs to support patients, with the goal of disseminating the learnings throughout levels of the organisation. Digital tools were frequently implemented across the sites for DM, where clinicians utilised a novel decision-support app (*Thinksulin*), as well as targeted online modules (e.g., insulin management, hypoglycaemia) to increase the skill of nurses and junior medical officers. Additional attention was focused on prioritising referrals to Diabetes Educators for all inpatients with DM, improving documentation and assessment of patients with DM in the emergency department, and the adoption of visualisation tools to chart blood glucose levels over time (rather than single point in time displays) to promote a holistic understanding of patient wellbeing.

### Main findings

Four capability development program theories initially hypothesised by the team were supported by evidence from interviews: *Making it Relevant, Investment in Quality Improvement, Turnover and Capability Loss*, and *Community-Wide Priority*. Two other initial program theories were incorporated into these four as they were refined. The four themes retained through analysis are broken down into contexts (C), mechanisms (M) and outcomes (O) below.

#### Theme 1: making it relevant

The final supported and refined CMO for this theme was:

Capability development activities and tools that address immediate clinical needs (C) open people’s eyes to delivering care differently (M), cultivating the knowledge, skill and confidence needed to deliver the evidence-based model of care (O).

It was clear that some capability training was welcomed for its relevance to daily care on the ward and a perceived deficit of knowledge, skills and confidence in spite of basic competence in insulin management. While such competency may be easily learnt from a textbook, there was clearly a need for greater capability in handling complex patients and rapidly changing situations. Assessing needs of the staff was a key strategy for programs without prescriptive models of care to follow.

We asked them in the survey what topics would you like [for in-service education for the initiative] and insulin was the top one. Types of insulin and when to withhold or when not because we’ve had incidents at the hospital, and there’s a lot of cases that aren’t reported, we’ve had hospital induced diabetes ketoacidosis because people have withheld long-acting insulin from patients with type one diabetes and we’ve sent them into DKA. [Project Lead07 IDDM District I]

There was evidence that capability development activities (e.g., CHF education workshops, training in smoking cessation programs, and use of glucose management tools) promoted clinician confidence and knowledge in managing patients according to best practice guidelines, and created motivation for further learning:

And from the feedback… the in-services… were really well received and a lot of people commented on how much more confident they felt with being able to care for the patient. They actually started asking for more in-depth in-services. [Project Lead12 CHF District C]

Capability development activities and tools addressed immediate clinical needs by targeting junior medical officers and nurses. Capability building in-service education and development and dissemination of high-quality, readily available tools (e.g., diabetes decision-making app) helped clinicians stay up to date with developments in best practice, and provided easy and flexible access to materials, including after hours:

Most of the time [it is the] junior staff that are looking after the people [with diabetes]… [The diabetes management tool] sort of guides you through the various scenarios, so have you thought about this? Have you checked this?… So, if you’ve never encountered this problem before… you feel more confident knowing that you’ve checked some things. You probably still call someone, but it is something people can use after hours… [Clinical Lead 31 IDDM District W].

Some clinicians reported experiencing barriers accessing capability development tools (e.g., online education modules) due to a lack of quarantined time for quality improvement activities. However, the motivation to learn how to deliver care differently (M) appeared to overcome time-based barriers to engaging with capability development materials among some clinicians:

No, we don’t get time to do it.… The training was really in depth, and it wasn’t just a matter of an hour… I think for most people it definitely took a few hours to do each module. [Project Lead 06 CHF District M]

#### Theme 2: investment in a learning culture and continuous quality improvement

The final supported and refined CMO for this theme was:

When the need for training is valued and participation is actively facilitated (C), senior management / executive team and clinicians understand each other’s responsibilities (M) and engagement with capability development resources increases (O).

Engaging with capability development activities increased understanding and feelings of support within and across teams, further promoting cooperation and collaboration within organisations:

But there’s a definite shift in relationships. So ward staff are feeling more supported by the heart failure services, and heart failure services were feeling more part of the ward, you know. There was definitely a change. [Project Lead07, DM District C]

The active facilitation of capability development activities also improved capacity when change efforts were implemented across levels of the organisation:

The more we are developing capability, the more we are making sure that is regularly drilled down to multiple levels of the organisation… So we are building capability and capacity across the district. [District Manager 27 all initiatives District W]

There was evidence that interest in capability development activities was particularly strong for staff caring for patients with complex conditions. At sites where a needs assessment was done, there was a clear gap in knowledge identified and staff were keen to communicate this need back to management. The provision of protected time to engage with capability development activities supported by senior management also appeared to strengthen engagement:

Yeah, they were given time to attend… because they all realise that there’s just not enough education around diabetes… so we were very well received. [District Manager 27 all initiatives District W]

However, attendance at educational workshops were affected by the culture of the department or team, suggesting differences in the facilitation and perceived value of capability development activities (C). Some were not given time to attend, leading staff to feel they needed to use their own (unpaid) time to participate. This lack of facilitation from management suggests a culture in which capability development was a lower priority than service provision, or other ward activities. On other wards there seemed to be a culture of not engaging in education; a culture that was driven by individual staff members or team leaders.

There are some wards that it was just part of their DNA. Every day they got an in-service and here’s [Dr X or Nurse Educator Y] in for the diabetes project. And they were really taking that on board. That was the expectation that they were allowed to have time… or time was invested for them to be able to take on education. Then other wards, it felt like that you were extracting teeth it was so much of an effort for them. [Project Lead 07 DM District I]

#### Theme 3: turnover and capability loss

The final supported and refined CMO hypothesis for this theme was:

Organisations with high staff turnover, particularly of key stakeholders for the initiative (C), leads to a loss of knowledge that prevents the workforce from learning from experience, a repetition of mistakes, a need to reinvent the wheel, and inhibition of continuous improvement (M). This results in a failure to produce critical mass of knowledge and skills within the organisation’s workforce to consistently deliver the evidence-based model of care (O).

Staff turnover, including rotation of junior medical officers, and changes in leadership led to a loss of knowledge and momentum to embed change. Capability development activities for the project seemed to be thought of as short term events that were replaced over time by new projects:

I would say [staff turnover] probably does inhibit improvement… New staff came and… it fell to the wayside as such, it wasn’t pushed. I guess it just never really got embedded… We were really enthusiastic, but then other things came out. [Clinical Lead 06 CHF District M]

New processes that were the responsibility of rotating junior medical officers who were constantly new to the system impeded desirable innovations moving to standard practice, fully integrated and supported by the system:

For example, the discharge supplement, that’s the hardest thing to implement, because you’ve got the doctors rotating every whatever. [Project Lead 07, IDDM District C]

#### Theme 4: community-wide priority

The final supported and refined CMO for this theme was:

Clinical roles that span sites (C) act as a conduit for standardisation of care processes across organisations (M) enabling a smoother transition for patients between sites (O).

There was evidence that clinical roles that worked across, or liaised between sites promoted standardised care based on the initiative, collaboration, and consistency across regions and enabled smooth transitions of patients moving from one facility to another:

[My] position was actually vacant for seven years… When I came on board, I’m the conduit, I guess, between the whole three districts, and we’re all working together now towards standardisation throughout (region X) in New South Wales. [Clinical Lead 17 DM District N]

Capability development included the funding of positions for specifically skilled staff (here a Respiratory specialist nurse). This helped achieve the aims of the COPD project to reduce readmissions but this interviewee thought changes were not sustained when the nurse was replaced by a remotely located nurse who was less accessible.

I felt like our respiratory nurse worked extremely hard and while she was doing that, our COPD patients definitely had a decrease in the amount of time that they would come into hospital. But she’s now moved on and we have someone from (a regional area), who, like, I think you have to refer to, so we probably haven’t seen a respiratory nurse now maybe for, you know, four or five months. [Clinical Lead 06 CHF District M]

### Guiding principles

Five guiding principles for designers or implementers or large-scale initiatives were distilled from the data:

#### Involve all levels of the health system to effectively implement large-scale capability development

While it is the clinicians on the wards that may be enacting change, their ownership and commitment to the initiatives is heavily influenced by macro- and meso-levels of the health system. State-wide agencies can contribute to large-scale change in capability development by rolling out initiatives that are promoted as a system priority, well researched, based on best available evidence and that are packaged with robust support for implementers. Within the local health districts, support from senior management and executive gives authorisation to all staff to embrace change initiatives, enables practical support to be given to clinicians (e.g., quarantined time for implementation activities, resources to employ new staff or buy equipment), and exerts influence to both validate effort and share accountability for progress. On the wards, senior clinicians and educators can foster a learning culture, encouraging engagement with and reflection on relevant audit data, and work in areas needing improvement.

#### Design capability development activities in a way that supports a learning culture

Teams and organisations that have a positive learning culture are known to be more effective in embedding change [27]. Characteristics of a learning culture include a commitment to continuous improvement, and the use of data to inform practice. Capability development requires more than the acquisition of specific knowledge, for example through an education module. It involves a complex interplay of learning, practicing, sometimes adapting, and reflecting to embed changes. Activities that actively target the gaps between initiatives’ goals and needs identified by clinical teams (e.g., acknowledged skills deficits), encourage engagement and ownership. Capability development activities that encourage ongoing opportunities to embed the new knowledge and understandings gained, and promotes ongoing reflection on improvement are more likely to sustain change and build the learning culture [17]. Key to creating and sustaining a learning culture is support from senior management to develop capability within work hours and incorporate these activities into routine work.

#### Plan capability development activities with staff turnover in mind

Rather than a single round of activities, plan to repeat them at regular intervals for new staff, or incorporate initial learnings into updates and ongoing events to ensure all staff’s capabilities are improving. Regular updates will maintain the currency and positive attitude of existing staff and reinforce the value of the initiative.

#### Increased capability should be distributed across teams to avoid bottlenecks in workflows and the risk of losing key staff

Individuals that hold particular capabilities can quickly become overwhelmed if they become the go-to person for that skill [28, 29] (e.g., only one clinician can perform a specialised assessment, or supervise a procedure). This potentially sets up bottlenecks for workflow as patients or other staff queue, in effect, for that capability. Developing capability across whole teams is a more efficient and sustainable approach and can also mitigate the risk of losing key staff. For leadership roles vulnerable to staff turnover or changing priorities, sharing the role and succession planning should be considered to maintain momentum of the initiative.

#### Foster cross-site collaboration to focus effort, reduce variation in practice and promote greater cohesion in patient care

Collaboration between sites can standardise practices, reduce effort, and increase engagement. Key to this are designated leadership or coordination roles that span sites and link up individual teams [30]. Whether the groups actually meet, or the cross-site coordinator acts as a go-between, collaboration can promote group problem solving, sharing of experiences and useful approaches, prevent duplication of effort, and keep the forward momentum of the initiative.

## Discussion

We undertook a realist evaluation to examine the role of strategies around developing capability in the implementation of three large-scale LBVC initiatives. Four of the hypothesised CMO statements were supported by the evidence. Guiding principles were distilled from the data on capability development strategies and complements another paper that defines overarching principles, distilled using the same methods from the entire data set across all the strategies [31]. 

Capability development activities included educational workshops, online modules, and local initiatives such as the development of decision support apps. These locally organised activities were seen to emerge in response to the top down, state-wide, large scale initiative of LBVC. Led by local educators, they were mostly well received and led to favourable outcomes. For instance, most participants described how capability development initiatives increased clinician knowledge, skill and confidence in delivering evidence-based care, particularly when targeted to areas identified as needing intervention. Capability development tools for DM management increased clinician understanding of insulin administration and promoted confidence in managing complex patients. In addition, participants described how coordination of capability development across sites led to a more cohesive service. There was also evidence that well run capability development activities promoted a learning culture, spurring emergent interest in further professional development and system improvements.

One CMO statement outlined the potential for negative outcomes when attempting to build a critical mass of skills in organisations with high staff turnover. Participants described the disruption caused by the loss of staff across stakeholder, executive and clinician levels as an impediment to the transition of initiative goals to usual practice. For example, short term, rotating junior medical officers were never long enough on the cardiac ward to progress the heart failure discharge summaries into standard practice, and the turnover of staff more generally, created a loss of momentum and critical mass of upskilled clinicians in the face of competing priorities. These findings are supported by those of previous studies, where change efforts in healthcare were hampered by a lack of continuity and repetition, missing opportunities to learn by experience [32]. 

Participants also discussed the importance of leadership and ownership in driving and sustaining change efforts gained through capability development. For instance, engaged clinical nurse educators surveyed staff to understand needs around diabetes management. Resulting skills training in diabetes led to a shared ownership of capability development activities around initiative priorities. These findings have support in the literature, where there is evidence that targeting activities to identified needs, and being supported by senior management helps to successfully integrate individual learnings into the broader team or organisation [17]. 

However, while a team’s capability can be enhanced through the appointment of uniquely skilled clinicians, the sustainability of change may be at risk. Key figures such as respiratory nurses or clinical educators across whole districts were able to standardise and coordinate high quality across regions. However, when a key person leaves, or a regional person is not known to all the sites, positive outcomes cannot be maintained. These findings highlight the importance of building sustainability into local systems through distributed responsibility and collective ownership to promote the dissemination of change efforts and maintain initiative momentum following turnover [6, 19]. In support of this, one participant discussed how individual and team ownership of process changes in heart failure care were necessary in order to establish them as part of business as usual. For example, establishing regular check-ins on initiative goals and including LBVC on the agenda at each meeting.

Similarly, while the content of the tools and activities appeared to be well-endorsed, several participants described difficulties around the provision of infrastructure and resources needed to support capability development initiatives. For example, a lack of quarantined time among clinicians to engage with materials and modules, staff turnover, and a lack of resources to standardise care efficiently and sustainably across organisations in the event of critical staff loss or absence. These barriers may indicate a lack of penetration and integration of activities into day-to-day practice to support the LBVC initiatives, and unstable momentum outside of key agents or drivers of change. These findings have support in the literature, where a survey of NHS nurses’ attitudes towards continuing professional development found that a lack of dedicated time to complete activities impeded uptake [8]. Specifically, nurses described a lack of staff to fill workforce gaps, lack of access to online modules after hours, and the expectation that staff would use personal time or annual leave to attend workshops. As a result, attention must be paid to providing enabling infrastructure and resources to support the uptake of capability development initiatives, including protected time and additional incentives, such as extrinsic rewards [17]. 

The promotion of a learning culture within wards, teams and districts was clearly associated with engagement with capability development initiatives. This was partially achieved through creating a mutual understanding of responsibilities between clinicians and senior management. The importance of strong managerial support for capability development activities has been evidenced in the literature, where learning cultures were encouraged by an understanding of the value of further training to improve the quality of care [8]. For example, in two studies on the development of capabilities among healthcare middle managers, contextual support provided through networking, assistance from upper management, and the creation of a “learning network”, allowed for the maintenance of change efforts in spite of staff turnover [32, 33]. Conversely, there is some evidence in the present findings and the literature that organisational culture may precede attitudes towards professional development activities. That is, staff attitudes to capability development activities may not reflect the acceptability of the initiatives themselves, but rather represent the broader cultural attitudes to learning and development [17]. In the present and previous work, clinician disinterest in capability development activities arose when the content did not adequately relate to their practice [8]. This appeared to be partially overcome by the tailoring of relevant activities to match clinical needs, as seen in the establishment of diabetes workshops and tools that were selected based on the nominated needs of clinicians.

Strengths and Limitations.

There are several notable strengths and limitations of the present work. Firstly, this study employed a realist methodology, which examined the interrelationships between contextual factors and key mechanisms driving the implementation of inpatient LBVC initiatives. Our understanding of perspectives across macro- and meso-levels may be limited on the topic of capability development strategies as interview responses were drawn from a relatively small sample, predominantly consisting of clinicians on the ground rather than executive perspectives. While the broader Australian context did not feature prominently, discussions of the impact of COVID-19, bushfires, floods and other natural disasters on care delivery were present in interviews across the project. These emergencies had significant effects on the health system and may have influenced capability development activities as well as the perspectives of respondents.

## Conclusions

This study employed a realist methodology to examine the circumstances in which the implementation of a large-scale value-based healthcare program to improve evidence-based care through capability development initiatives. In line with previous findings, capability development activities promoted the creation of a learning culture, greater cohesion within and between teams, and increased standardisation of care across sites. However, the provision of resources to support care improvement, as well as the role of team dynamics and staff turnover were key contextual factors that affected the translation of initiative goals into desired outcomes. Taken together, these findings may be of particular benefit for executives, stakeholders and managers seeking to develop capability in their organisations for the implementation of large-scale quality improvement initiatives.

### Electronic supplementary material

Below is the link to the electronic supplementary material.

Supplementary Material 1

Supplementary Material 2

## Data Availability

De-identified data (e.g., coding structure and iterations of the CMO statements) not reported on in this paper are available on reasonable request from the corresponding author. Raw data including full transcripts of interviews are not available as it is not possible to de-identify fully. Ethics requires full confidentiality.

## Abbreviations

CHF: Chronic heart failure
COPD: Chronic obstructive pulmonary disease
CMO: Context-Mechanism-Outcome
DM: Diabetes mellitus
LBVC: Leading Better Value Care
NSW: New South Wales
