# Supplementary material for: Implementation of large, multi-site hospital interventions: a realist evaluation of strategies for developing capability
Source: BMC Health Serv Res. 2024 Mar 6;24:303. doi: 10.1186/s12913-024-10721-w (PMC10918928; doi:10.1186/s12913-024-10721-w)
Supplement: Supplementary file 1 — Supplementary Material 1 [file 12913_2024_10721_MOESM1_ESM.docx]

| **Capability development** | | | | |
| --- | --- | --- | --- | --- |
| **Strategies** | | | **Initiatives** | |
| Implementation re-design workshops | | | LBVC | |
| Health behaviour change coaching | | | OACCP, ORP | |
| Audit and feedback -> Insulin monitoring **app**, JMO e-learning module for insulin prescription | | | IMDM | |
| **Context** | **Mechanism** | **Outcome** | | **Questions** |
| 1a. All sites and clinicians can access relevant, iterative and ongoing capability development that addresses immediate knowledge and skill needs, while incorporating practical application through knowledge-to-action processes (including funding for rural practitioners). | 1a. Creating a shared mental model by opening people’s eyes to seeing things differently and what is important, **generating self-esteem, confidence**, and focusing attention on the right things. | 1a.  **O1:** Knowledge, skill, and replicating success:  Knowledge and skills required to deliver evidence-based model of care cultivated over time, throughout the organisation.  **O2:** The success of initiatives at single sites can be replicated across multiple sites at scale. | | I’d first like to talk about the capability development components for LBVC and the role that played in implementation of the program. We’ve got a few ideas about that, some of which I’ll introduce later, but can you tell me initially what made up the capability development activities locally?  [prompt examples]   - How did that help to drive change? (M) - What did that lead to? (O) - How did you find out about that? - Was that the case for everyone? (C)   Do you think that clinician’s knowledge and skills have improved as a result of these activities? (O)   - Did that contribute to the success of the initiative/s? (O) - How do you know? - Do you think it was effective in driving change because the staff were all on the same page? (M) - Did it build confidence? - Did it build self-esteem? (M) - Did it focus clinician’s attention? (M) - How was the training made relevant and accessible to clinicians at your site? (C) - Was it because the training addressed immediate need? (C) |
| 1b. High staff turnover:  Organisations with high staff turnover, particularly of key stakeholders for the initiative. | 1b. Knowledge loss:  Loss of knowledge prevents workforce from learning from experience, repeating mistakes, reinventing the wheel, and inhibiting continuous improvement. | 1b. Incoherence:  Failure to produce critical mass of knowledge and skills within organisations workforce to consistently deliver evidence-based model of care. | | Was there enough knowledge and skill developed? (O)   - Why was this? (C) - What did that lead to? (M) - Could you give me an example? - Do you have any other examples? - Were there any examples where high staff turnover led to repeated mistakes? Reinventing the wheel? (M) - How was it overcome, if it was? |
| 2a. Investment in quality improvement:  Clinical staff are given rostered time for initiatives and capability development activity, which includes QI tools and coaching in addition to clinical skill and knowledge building. | 2a. Understanding where staff are “coming from”:  Staff feel respected and can see investment in their development, rather than adding to workloads. This leads to a perspective shift from viewing projects in isolation to incorporating within existing quality improvement structures and processes. | 2a. Adoption of QI culture:  Ownership of the initiative shifts from project personnel to operational leaders, managers, and frontline staff. | | Do you think your site adopted a quality improvement culture in relation to the LBVC initiatives? (O)   - What does that look like? (O) - How did that happen there? What came before? (M) - Were staff given rostered time for the initiatives? (C) - Did staff have enough tools or activities? (C) - How did that help? (M) |
| 2b. Lack of co-development in design of training with mostly didactic learning approach. | 2b. Capability development activities are viewed as imposition of clinicians and another task in their already busy days. | 2b. Knowledge and skills development does not translate into practice change. | | Did the knowledge and skills development ever not translate into practice change? (O)   - Why was that? (M) - Which initiatives? (C) - Were the activities ever considered an impost on clinician’s time? (M) - Which activities? (C) - Who was involved in developing the training? (C) - Did it match the clinician’s needs? (C) - Were clinicians involved in the development? (C) |
| 3a. Peer interaction:  Capability development opportunity includes peer interaction and is fully funded (including transport for rural practitioners) for both internal and external staff, to integrate primary care. | 3a. Community-wide priority:  The initiative is considered a whole of community issue to be addressed, and a collaborative model develops between individuals across different organisations. | 3a. Reach and scale:  The success of initiatives at single sites can be replicated across multiple sites at scale. | | Were the LBVC initiatives a community-wide priority in your LHD? (M)   - How did they do that? (M) - What did that lead to? (O) - Did it enhance the reach and scale of the program? (O) - Why did people outside the hospital get on board? (C) - How did [answer] lead to a community wide priority? - Were there peer interaction activities? (C) |
| 3b. Capability development does not include peer learning, mentoring and ongoing community of practice. | 3b. Network information flow block:  Lack of information flow from within and between organisations. | 3b. Local successes are not able to be adapted to other contexts due to lack of information flow. | | Did you share or receive information about what worked or didn’t work from other sites? (M)   - What did this lead to? (O) - Did the lack of information flow prevent success at your site? (O) - What did you miss out on? (what information?) (C) - Was there peer learning, mentoring, ongoing communities of practice? (C) |
| Opener:   1. Thank you for agreeing to participate today. 2. I’ve started recording, I just wanted to check you have read and understood the information form and are happy to participate today? 3. Purpose of this interview is to understand more about the implementation of the LBVC program, so this is not an evaluation and we’re not evaluating you personally. We’re trying to understand how the various implementation strategies led to either planned or unplanned changes throughout the system. 4. Have you participated in a research interview before? The interview style we are using is a bit different to what people might be used to, I’ll be asking a lot of probing questions, such as ‘Can you give me an example? How do you know x or y” – but just to reassure you that when you name specific people or sites that it is all confidential. 5. Can you briefly describe your role in relation to the LBVC program? | | | | |

**References**

1. Atkinson, J., et al., *tDrivers of large-scale change in complex health systems: a rapid.* 2013.

2. Rycroft-Malone, J., et al., *Improving skills and care standards in the support workforce for older people: a realist synthesis of workforce development interventions.* 2016.

3. Graham, A.C. and S. McAleer, *An overview of realist evaluation for simulation-based education.* Advances in Simulation, 2018. **3**(1): p. 13.
